# Preparation of *Albatrellus ovinus* β-Glucan Microparticles with Dectin-1a Binding Properties

**DOI:** 10.1021/acsabm.3c00071

**Published:** 2023-04-19

**Authors:** Christiane F. Ellefsen, Anna-Maria Struzek, Regina Scherließ, Marianne Hiorth, Anne Berit C. Samuelsen

**Affiliations:** † Department of Pharmacy, Faculty of Mathematics and Natural Sciences, University of Oslo, NO-0316 Oslo, Norway; ‡ Department of Pharmaceutics and Biopharmaceutics, Kiel University, D-24118 Kiel, Germany; § Priority Research Area Kiel Nano, Surface and Interface Sciences (KiNSIS), Kiel University, D-24118 Kiel, Germany

**Keywords:** milling, precipitation, mushroom β-glucan, particulate β-glucan, dectin-1a

## Abstract

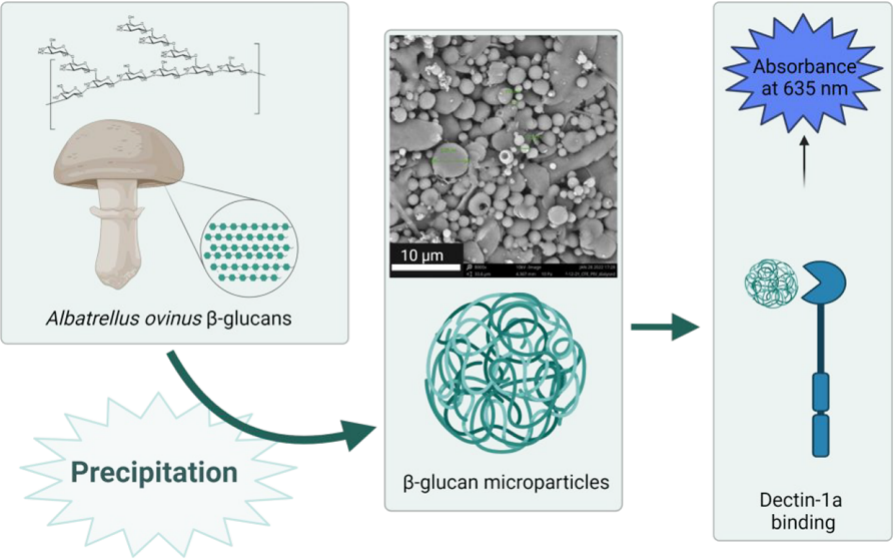

Fungal β-glucans are compounds with the potential to activate the innate immune system, in part through binding to the receptor dectin-1. In the present study, small-scale methods for preparing dectin-1a binding microparticles from *Albatrellus ovinus* alkali-soluble β-glucans were investigated. Mechanical milling was time-consuming and yielded large particles with wide size distributions. Precipitation was more successful: the β-glucan was dissolved in 1 M NaOH, diluted, and precipitated in 1:1 mol equiv HCl. This yielded particles in sizes ranging from 0.5–2 μm. The dectin-1a binding activity was determined using HEK-Blue reporter cells. The prepared particles were able to bind to dectin-1a to the same extent as baker’s-yeast-derived β-glucan particles. The precipitation method was convenient as a quick method for small-scale preparation of β-glucan microparticle dispersions from mushroom β-glucans.

## 1 Introduction

Fungal β-glucans have the potential to modulate the immune system through binding to pattern recognition receptors (PRRs) expressed by innate immune cells.^1^ This is due to structural similarities between fungal β-glucans and structures found on the cell surfaces of pathogenic fungi.^2^ The fungal β-glucans typically consist of a β-(1 → 3)-linked d-glucopyranosyl (Glc*p*) backbone with branches of β-(1 → 6)-linked d-Glc*p* residues situated at *C*-6 on the backbone. In addition, there are (1 → 6)-linked β-d-glucans having side chains linked at *C*-3. β-glucans with different linkage patterns have been isolated from different sources, and the nature of the backbone as well as the frequency and length of side chains may vary depending on the fungal species, as reviewed by Synytsya and Novák.^3^ This leads to variations in properties such as water solubility and affinity to PRRs.^4^ In general, linear (1 → 3)-β-d-glucans are insoluble in water due to extensive intra- and intermolecular hydrogen bonding, whereas for branched β-(1 → 3),(1 → 6)-glucans, the water solubility increases with the frequency and distribution of side chains.^5^

Several PRRs have been identified as β-glucan-binding receptors: the c-type lectin receptor dectin-1,^2^ complement receptor 3 (CR3; also known as CD11b/CD18),^6,7^ lactosylceramide,^8^ and selected scavenger receptors.^9^ Furthermore, several Toll-like receptors (TLRs), including TLR2 and TLR4, may be involved.^10−13^ In some cases, activation depends on simultaneous binding of different types of PRRs by the ligand. Details on this and the activation pathways involved for the individual PRRs are thoroughly discussed in a recent review.^14^ Additionally, the activation of PRRs has been reported to be affected by β-glucan solubility. For instance, particulate β-glucans are able to polarize macrophages into the proinflammatory M1 phenotype through dectin-1 activation.^15,16^ Soluble β-glucans, on the other hand, are able to activate human dectin-1 but not murine dectin-1.^17^ The reason for this seems to be related to the presence of two different isoforms of dectin-1: the full-length dectin-1a and stalk-less dectin-1b; both types are expressed by human and murine immune cells but at different concentrations.^18−20^ The antagonistic activity of soluble β-glucans has been shown to be limited to the dectin-1b isoform.^21^ There is still limited knowledge about the functional differences between the two dectin-1 isoforms.^20^ However, it has been shown that dectin-1a can be activated by both soluble and particulate β-glucans, whereas dectin-1b is only activated by particulate β-glucans.^12^ It has been suggested that this is caused by dual binding of TLRs and dectin-1a or dectin-1b for the activation to occur. Soluble β-glucan molecules are unable to interact simultaneously with both the stalk-less dectin-1b and TLR receptors; thus, the antagonistic activity is achieved.^13,14^

Knowledge on how various properties of the particulate β-glucans, such as particle size and morphology or differences in polymer structure and conformation, affect the interaction with dectin-1 is still limited.^22^ In order to investigate this further and in order to compare the activity of water-insoluble β-glucans from different fungal sources, there is need to develop a simple and reliable method to prepare particles from this type of material.

Previously, it has been shown that β-glucan nanoparticles of 355 nm were able to induce production of proinflammatory cytokines from human peripheral blood mononuclear cells, whereas nanoparticles of 130 nm did not have this activity.^23^ In addition, microparticles of 2–3 μm seem to be optimal for phagocytosis by macrophages.^24^ Thus, the desired particle size to be prepared should be within the 0.3–3 μm size range.

β-glucan-based particles have previously been prepared from baker’s yeast (*Saccharomyces cerevisiae*) by washing yeast cells with solvents of varying pH and polarity, at varying temperatures and pressure.^25−27^ This leaves behind a porous β-glucan-rich cell wall shell of 2–4 μm diameter that functions as a particle. Other methods are based on dissolving the purified β-glucan followed by precipitation through changes in the solvent system that reduce the solubility of β-glucan. For example, particles of 0.3–1 μm were prepared from zymosan, a yeast-derived β-glucan preparation, by dissolution in DMSO and precipitation with trifluoroacetic acid (TFA).^28^ Cellulose particles have been prepared similarly by precipitation in EtOH.^29^ Another approach is to break down solid material into smaller particles mechanically. Particles of around 80 nm have been prepared by ball milling of a β-glucan-containing yeast extract.^30^ Whether these methods translate to β-glucans from mushrooms has, to our knowledge, not been investigated yet.

The aim of the current work was to develop a method for preparation of β-glucan microparticles within the size range of 0.3–3 μm, from water-insoluble mushroom β-glucans. The size and morphology of the particles were investigated along with initial assessment of their ability to bind to the human dectin-1a receptor.

## 2 Experimental Section

### 2.1 β-Glucans

A sheep polypore mushroom (*Albatrellus ovinus*) β-glucan-rich fraction was isolated previously as described in Samuelsen et al.^31^ In short, mushroom fruiting bodies were lyophilized and ground to a fine powder using a blender. This powder was then subjected to a sequence of extractions with dichloromethane, EtOH, and H_2_O. In the end, alkaline extraction was performed twice with 1 M NaOH with 0.135 M NaBH_4_ at 100 °C under reflux. The extract was precipitated with three volumes of 96% (v/v) EtOH at 4 °C overnight, yielding the alkali extract AAo, which was further separated into the water-soluble fraction AAoSw and water-insoluble fraction AAoIw. AAoIw, the β-glucan-rich fraction, was used in this study as a test material for method development.

Baker’s yeast (*S. cerevisiae*)-derived particulate β-glucans zymosan A (ZymA) and zymosan depleted (ZymD) were purchased from Sigma-Aldrich (St. Louis, MO, USA) and InvivoGen (www.invivogen.com, Toulouse, France), respectively. The *S. cerevisiae* glucan Wellmune WGP Dispersible powder (BG-P; Lot 09240–04; Biothera, Eagan, MN, USA) and partially water-soluble β-glucan (Wellmune, WGP Soluble powder, Lot 09297–02, Biothera) were provided as a gift by Immitec, Tønsberg, Norway. From the latter material, a water-soluble fraction (BG-S) had been obtained previously as described by ref (16). M-Gard (M-G), another *S. cerevisiae* particulate β-glucan preparation, was provided as a gift from Biotec BetaGlucans AS (Tromsø, Norway). The soluble β-glucan laminarin, from the algae *Laminaria digitatae*, was purchased from Sigma-Aldrich.

The particle size and size distributions of BG-P and M-G were obtained by laser diffraction analyses using a HELOS/KR-RODOS (Sympatec GmbH, Clausthal-Zellerfeld, Germany) with Windox 5.8.2.0 software (Sympatec). Measurements were made in triplicates.

### 2.2 Particle Preparation by Mechanical Methods

#### 2.2.1 Ultra-Turrax Milling

The water-insoluble fraction AAoIw (18 mg) was suspended in 12 mL of Milli-Q water. The suspension was milled with a T25 digital Ultra-Turrax (Ika, Staufen, Germany) equipped with an S 25 N-8-G-ST tip (Ika), in 15 min intervals at 25 000 rpm with ice cooling, for a total of 10 h. The decrease in particle size was tracked by laser diffraction measurements as described below. The final suspension was centrifuged (3500 rpm, 15 min), the supernatant was removed, and the pellet was washed with 15 mL of 96% (v/v) EtOH and centrifuged. The pellet was dried in air and designated UT-M (Ultra-Turrax-Milled).

#### 2.2.2 Ball Milling

AAoIw (50 mg) was ground in a ball mill (Retsch MM400, Haan, Germany) by placing the dry material in a 25 mL stainless steel grinding jar and adding 5 g of grinding balls (3 mm, stainless steel). The instrument was set to a vibrational frequency of 25 Hz, and the sample was milled in 30 min intervals. After 40 min, 100 min, and 8 h of milling, the resulting particles designated B-M (ball-milled), were collected with 15 mL of 96% (v/v) EtOH and centrifuged (3500 rpm, 15 min). The pellet was resuspended in EtOH for size measuring by laser diffraction and air-dried for SEM imaging.

### 2.3 Particle Preparation by Precipitation

#### 2.3.1 Precipitation

Powdered AAoIw (25 mg) was suspended in 1 M NaOH (5 mL) and dissolved upon stirring and heating to 60 °C for 15 min. The glucan solution was cooled to room temperature and added dropwise to stirring solutions of 96% (v/v) EtOH, 70% (v/v) EtOH, H_2_O, or dilute HCl, resulting in the samples P01–P04 (Table 1). The samples PI–PV were made by dropwise (1 drop/s) addition of 10 mL of diluted glucan/NaOH solution into 2 vol of 1:1 mol equiv of HCl under constant stirring (Table 1). Three batches of samples were made using the same precipitation conditions, respectively.

**Table 1:** Experimental Setup of the Precipitation Experiments

	Solvent concentrations			Antisolvent concentrations		
Sample	NaOH (M)	AAoIw (mg/mL)	pH	EtOH (% v/v)	HCl (M)	pH
P01	1	5	14.0	96	-	7.0
P02	1	5	14.0	70	-	7.0
P03	1	5	14.0	0^a^	-	7.0
P04	1	5	14.0	-	1	0.0
PI	0.1	0.5	13.0	-	0.05	1.3
PII	0.075	0.38	12.9	-	0.038	1.4
PIII	0.05	0.25	12.7	-	0.025	1.6
PIV	0.025	0.13	12.4	-	0.013	1.9
PV	0.01	0.05	12.0	-	0.005	2.3

a 100% Milli-Q water.

#### 2.3.2 Dialysis

Samples PI–PV were dialyzed using SpectraPor Dialysis membrane Mw cutoff 3.5 kDa (SpectraPor Dialysis membrane, Spectrum Chemical Mfg. Corp., New Brunswick, NJ, USA) for removal of NaCl formed during precipitation. Dialysis was run for 5 days against Milli-Q water that was changed daily until a negative chloride test with 1% (w/v) aqueous solution of AgNO_3_.

#### 2.3.3 Resuspension and Sonication

Samples PI–PV (1.0–1.5 mg) were resuspended in Milli-Q water (1.0–1.5 mL) and sonicated with a Vibra cell VCX 130 W ultrasonic processor (Sonics & Materials Inc., Newtown, CT, USA) equipped with a 3 mm tapered microtip (Sonics & Materials Inc.) for 0, 5, 10, 20, or 40 s at 40% amplitude.

### 2.4 Characterization

The samples B-M, UT-M, and PI–PV were characterized either by laser diffraction or by dynamic light scattering (DLS) and scanning electron microscopy (SEM) as described in Sections 2.4.1–2.4.3. Pretreatment of the samples prior to each analysis is indicated in Table 2.

**Table 2:** Preparation Methods and Pretreatment of Samples in Combination with the Utilized Characterization Methods, Including the Dectin-1a Binding Assay^a^

Sample designation	Preparation method	Laser diffraction	DLS measurement	SEM imaging	Dectin-1a binding assay
UT-M	Ultra-Turrax-milled	+	-	+	-
B-M	Ball-milled	+	-	+	-
P	Freshly precipitated	-	+	-	-
P+L	Precipitated and lyophilized	-	+	-	-
P+L+S	Precipitated, lyophilized, and sonicated (5–40 s)	-	+	-	+
D	Dialyzed	-	+	-	-
D+L	Dialyzed and lyophilized	-	+	+	-
D+L+S	Dialyzed, lyophilized, and sonicated (40 s)	-	+	-	-

a DLS - dynamic light scattering; SEM - scanning electron microscopy. For SEM imaging, the lyophilized samples were imaged directly, while for DLS and dectin-1a binding assays, the samples were resuspended at 1 mg/mL before analysis.

#### 2.4.1 Laser Diffraction

Particle size and size distributions of UT-M and B-M with 8 h milling time were determined by laser diffraction using a HELOS-CUVETTE 6 R1 (Sympatec). The samples were dispersed in water (1 mL) in a 6 mL cuvette, diluted with water (5 mL), and analyzed using Windox 5.8.0.0 software (Sympatec), applying Fraunhofer theory. The particle size distribution of B-M, 40 and 100 min milling times, were determined by laser diffraction using HELOS/KR-QUIXEL R3 (Sympatec). The pellet was resuspended in 1 mL of 96% (v/v) EtOH, diluted with 250 mL of distilled water, and sonicated for 120 s. The analysis was performed in triplicate at ambient temperature. The particle sizes are presented as the distribution density and sum of distribution. The 10×, 50×, and 90× were taken from the sum of distribution, which represent the upper size limit of 10, 50, and 90% of the particles.

#### 2.4.2 Dynamic Light Scattering (DLS)

The samples PI–PV were analyzed by DLS after pretreatment as specified in Table 2. Lyophilized samples were resuspended 1 mg/mL in Milli-Q water prior to the analysis. Experiments were performed using a Zetasizer Nano ZS (Malvern Instruments Ltd., Worcestershire, UK) using backscatter detection at 173° at 25 °C. The refractive index and viscosity were set to those of pure water, while the material setting was set to polystyrene latex particles.^32^ Measurements were performed with temperature equilibration times of 300 s. The particle sizes were measured using the cumulant fit model from which the *z*-average and polydispersity indexes (PDIs) were derived as well as the general-purpose fitting model that yielded the peak size.

The cumulant fit analysis is the standardized method for obtaining DLS size measurements. However, when measuring polydisperse or heterogeneous samples, the cumulant method is not always suitable.^33^ In such instances, the peak size, or peak distribution, can be a better choice. This value is derived from a general-purpose distribution algorithm, which uses a larger part of the correlation curve to obtain a distribution of size peaks, each with their mean diameter size and size range. Certain studies have shown that the peak size can be a good approximation of the particle diameter.^32^ Herein, the presented peak size accounts for >90% of the total particle population by intensity, as calculated by the DLS software. As this method only shows particles within a specific size range, any larger agglomerates will not be detected. The cumulant method, on the other hand, is sensitive to larger agglomerates and will thus be affected if any such are present.

#### 2.4.3 Scanning Electron Microscopy (SEM)

Dialyzed and lyophilized PI–PV were visualized using SEM. The samples were sputtered with gold utilizing a Bal-Tech SCP 005 Sputter Coater (Bal-Tech AG, Lichtenstein) for 40 s. SEM images were taken with a Phenom World XL (Thermo-Fisher Scientific Inc., Waltham, MA, USA) at a working voltage of 10 kV.

#### 2.4.4 Congo Red Assay

The Congo Red assay for determination of the triple helix conformation was adapted from Ogawa et al.^34^ AAoIW was dissolved in 1 M NaOH at 5 mg/mL (AAoIw low) and 10 mg/mL (AAoIw high) by heating at 60 °C for 15 min or until completely dissolved. The glucan solutions were diluted with Milli-Q water to obtain the solvent concentrations of PI–PV (Table 1), along with a series of dilutions with the same NaOH concentrations but double the glucan concentrations. Additionally, each solution was diluted to obtain a 0.3 M NaOH concentration.

Each glucan solution (275 μL) was added to a microtiter plate in triplicates. Laminarin and BG-S, 1 mg/mL in 0.05 M NaOH, were used as controls. NaOH at each of the concentrations listed in Table 1 and 0.3 M NaOH were used as blanks. Then, 5 μL of 3.2 μM Congo Red (disodium 4-amino-3-[4-[4-(1-amino-4-sulfonatonaphthalen-2-yl)diazenylphenyl]phenyl]diazenyl-naphthalene-1-sulfonate; VWR International AS, Oslo, Norway) in 0.05 M NaOH was added to each well. After 16 h of resting, the plate was shaken for 3 min before absorbance reading between 350–650 nm, with 5 nm increments. A shift in *A*_max_ compared to the *A*_max_ of the blanks were interpreted as indications of triple helix conformation.

### 2.5 Dectin-1a Binding Assay

HEK-Blue hDectin-1a cells and HEK-Blue Null1-v cells (InvivoGen) were cultured in Dulbecco’s modified Eagles medium (DMEM; Gibco, Bleiswijk, The Netherlands) containing 2 mM l-glutamine and supplemented with 10% (v/v) fetal bovine serum (FBS), 100 U/mL penicillin, 100 μg/mL streptomycin, and 100 μg/mL Normocin (InvivoGen), and either 100 μg/mL Zeocin (InvivoGen) or 1 μg/mL puromycin (InvivoGen) and 1× HEK-Blue CLR Selection (InvivoGen) as specified by the manufacturer.

The samples PI–PV were suspended in Milli-Q water and sonicated (40 s) before being added in three concentrations (25, 2.5, and 0.25 μg/mL) to a 96-well plate. The concentration was based upon the content of β-glucan in the samples, as they also contained NaCl. The relative concentration of glucan and NaCl was 1:11.5. This entailed that cells treated with 25 μg/mL glucan were simultaneously treated with 288 μg/mL, or 4.9 mM, NaCl. A control experiment was performed by treating the cells with 1.5 mg/mL NaCl. The results showed that this did not lead to NF-κB activation nor did it cause decreased cell viability as detected by the MTT assay (unpublished results). This is also in accordance with reported toxicity levels of NaCl in different *in vitro* systems.^35^ Additionally, ZymA (10 and 1 μg/mL), ZymD (10 and 1 μg/mL), lipopolysaccharide (LPS; 100 μg/mL) from *Escherichia coli* (055:B5, Sigma-Aldrich), the lipopeptide Pam2CSK4 (100 μg/mL; InvivoGen), and the yeast-derived β-glucan products BG-P (100, 10, and 1 μg/mL), BG-S (100, 10, and 1 μg/mL), and M-G (100, 10, and 1 μg/mL) were added to the plate. Tumor necrosis factor-α (TNF-α; 100 and 10 ng/mL; Thermo-Fisher) was added as a positive control with the HEK-Blue Null1-v cells. The volumes added were 20 μL for all samples and controls. Cells were harvested with PBS (Gibco) and diluted with HEK-Blue detection medium (InvivoGen) to a concentration of 280 000 cells/mL. Cell suspension (180 μL) was added to each well of the plate, equivalent to 50 000 cells/well. The plate was incubated for 16 h in a humid incubator with 5% (v/v) CO_2_ at 37 °C. Cell supernatants (150 μL) were transferred to a new 96-well plate, and the absorbance was measured at 635 nm to register the release of secreted embryonic alkaline phosphatase (SEAP), as a sign of activation of nuclear factor κB (NF-κB) pathways (www.invivogen.com). All samples were assessed in triplicates, and the assay was repeated three times in total.

### 2.6 Statistical Analysis

Statistical significance was determined by a one-way ANOVA applying Dunnett’s multiple comparisons test using GraphPad Prism v.9.3.1 (GraphPad Software, San Diego, CA, USA). Data were expressed as the mean ± the standard deviation. *P* < 0.05 was considered statistically significant.

## 3 Results and Discussion

### 3.1 Mechanical Methods

**Figure 1:**
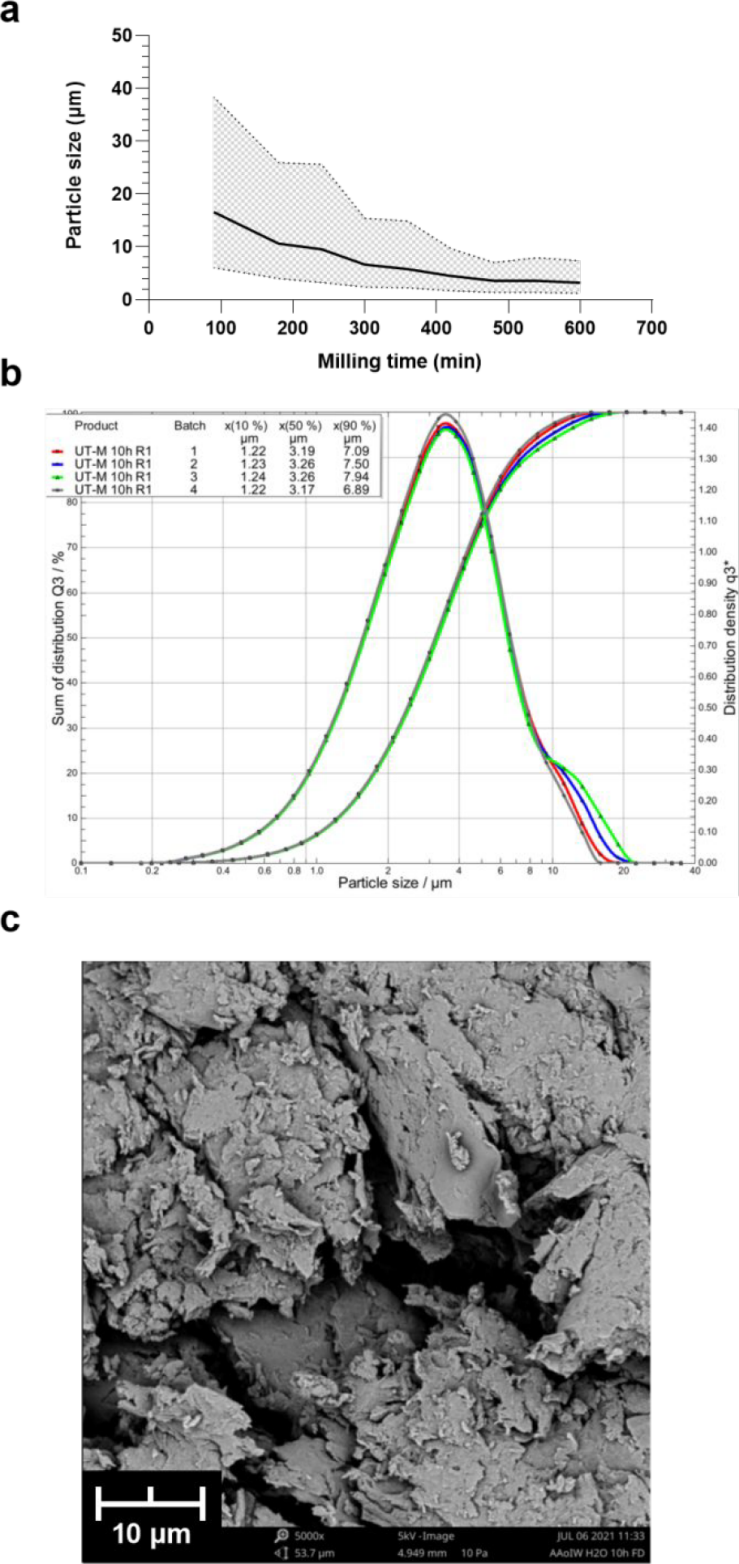
Ultra-Turrax milling (UT-M). (a) Decrease in average particle size (μm) and size range corresponding to 80% of the particle population, with increased time of milling, measured by laser diffraction. The 10×, 50×, and 90× were adopted from the sum of distribution and visualized as the lower limit, mean, and upper limit of the size. (b) Size distribution of particles after 10 h of Ultra-Turrax milling in water. (c) SEM image of sample after 10 h milling.

As a mechanical method for reducing the β-glucan particle sizes, milling with an Ultra-Turrax with H_2_O as the dispersing agent was attempted, resulting in UT-M. The size average of UT-M seemed to stabilize at around 3 μm after 9–10 h of total milling time, with a relatively narrow size range (Figure 1a,b). Approximately 50% of the particles was thus in the desired size range. SEM imaging (Figure 1c) illustrated the irregular, jagged shapes of the particles formed by this method.

**Figure 2:**
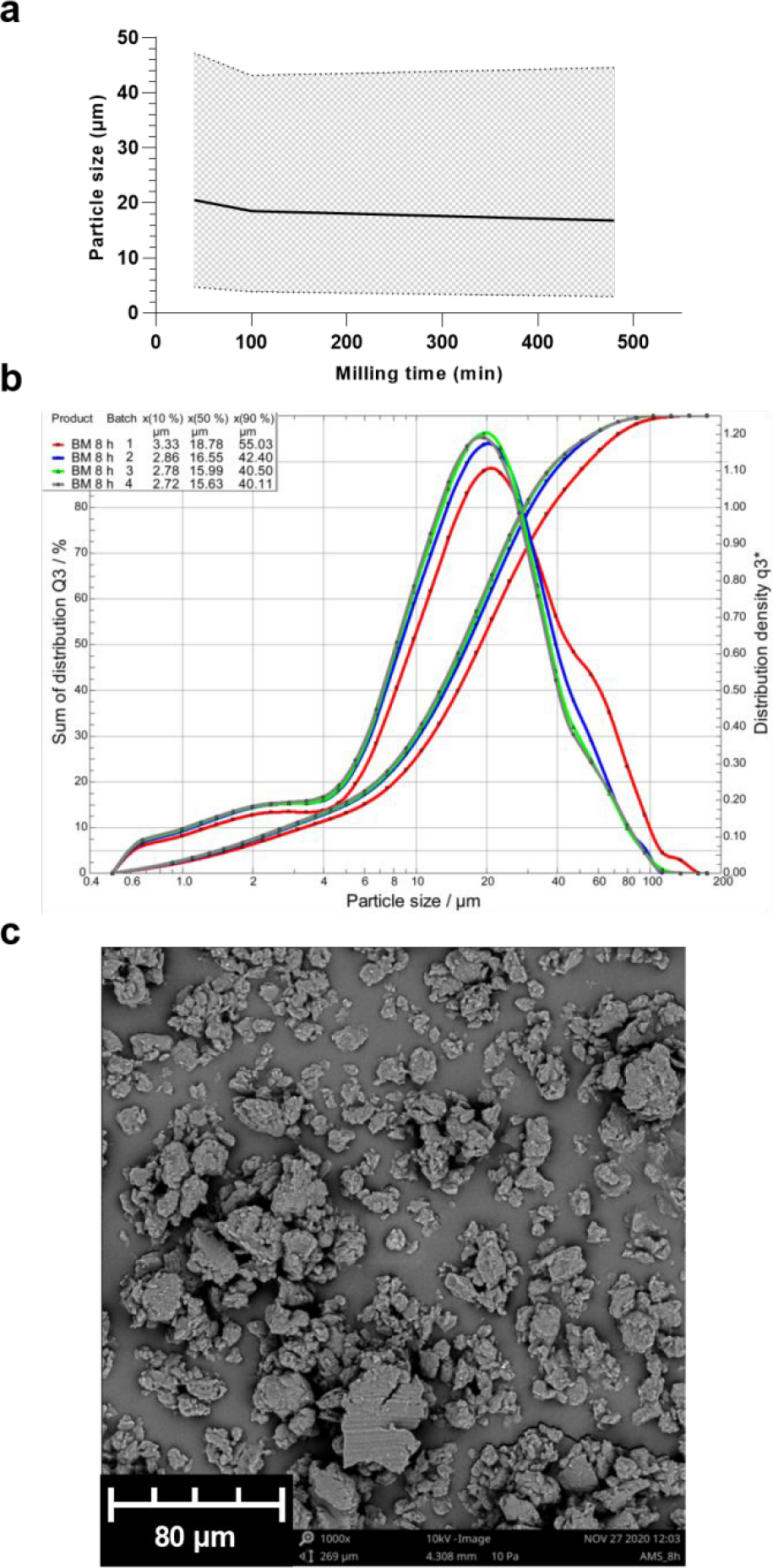
Ball milling (B-M). (a) Decrease in average particle size (μm) and size range corresponding to 80% of the particle population, with increased time of dry milling, measured by laser diffraction. The 10×, 50×, and 90× were adopted from the sum of distribution and visualized as the lower limit, mean, and upper limit of the size. (b) Size distribution of particles after 8 h of dry milling. (c) SEM image of the sample after 8 h milling.

Although the method yielded particles within the desired size range (0.3–3 μm), the method was associated with major drawbacks due to time consumption, massive strain on the instrument, and its inability to handle larger amounts of sample. Thus, this method was not investigated further. Ball milling can handle larger amounts of material and can be performed in a dry state. After 8 h of ball milling, the particle size seemed to stagnate at around 18 μm, with a large size range of 3–45 μm, as shown in Figure 2. Only about 10% of the particles were within the desired size range (Figure 2b). This is in discordance with previously reported <100 nm particle sizes achieved by 35 min of dry ball milling of oat, barley, and yeast β-glucans.^30^ The particles obtained by this method were of irregular shapes, as shown by SEM imaging (Figure 2c). Due to the time consumption and the inability of this method to yield the desired particle sizes, dry ball milling was also deemed an unsuitable method.

### 3.2 Precipitation

Different precipitation conditions were tested on the AAoIw sample dissolved in 1 M NaOH. Using 96% or 70% EtOH as the antisolvent resulted in flaky, inhomogeneous suspensions, and precipitation of β-glucan material with EtOH was therefore considered unsuitable. Precipitation did not occur when the β-glucan solution was added to Milli-Q water. However, adding the β-glucan solution to dilute HCl led to the formation of particles. As individual particles were discernible to the naked eye, they were likely larger than the desired particle size. With the intention to decrease the particle size, precipitations were performed with a series of dilutions of both the glucan and HCl solutions (Table 1).

#### 3.2.1 Particle Size and PDI of Freshly Precipitated Samples and Concentration Effect

**Figure 3:**
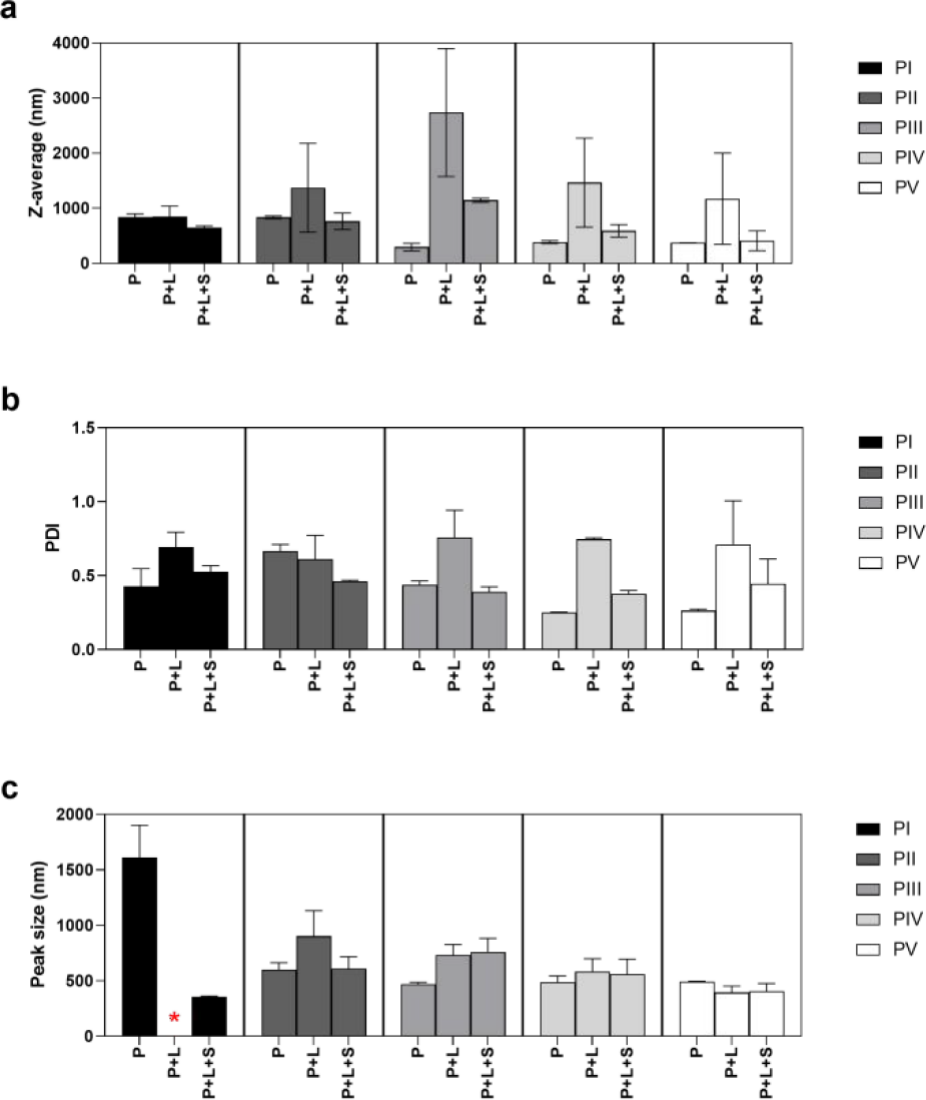
Particle sizes and size distributions of precipitated samples. (a) *z*-Average, (b) polydispersity index (PDI), and (c) main peak size of freshly precipitated (P), lyophilized and resuspended (P+L), or lyophilized, resuspended, and sonicated for 40 s (P+L+S) samples. Error bars represent the SD of a minimum of two series of measurements. * inaccurate values due to sedimentation.

Freshly precipitated samples (PI–PV) were found to be relatively heterogeneous in terms of particle sizes and size distributions (Figure 3). The *z*-average varied from 290–840 nm, while the PDIs varied from 0.25–0.67. The main peak sizes were between 470–600 nm, except for PI, which had a peak size outside the size range of the method. The differences in measured z-average and peak size indicated the presence of larger agglomerates and sample inhomogeneity. Both particle size and PDI decreased with the decrease in concentrations used during the precipitation. In order to achieve higher concentration of the samples and thereby more reliable size measurements, the samples were lyophilized and resuspended to a fixed concentration before size measurements.

#### 3.2.2 Effect of Lyophilization and Sonication

**Figure 4:**
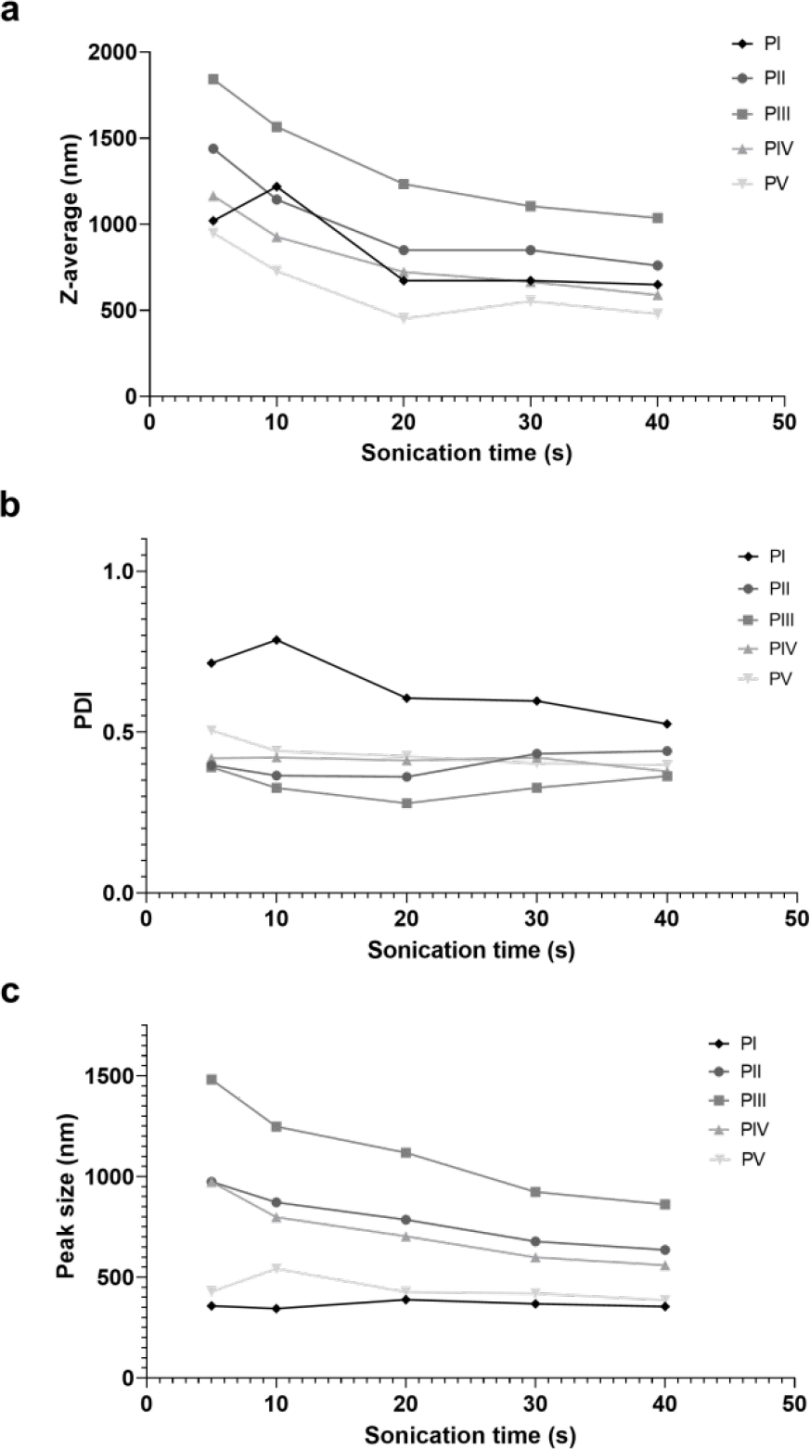
Sonication effects on particle properties. Change in (a) *z*-average (nm), (b) PDI, and (c) peak size (nm) with increasing sonication time (s) of precipitated samples PI, PII, PIII, PIV, and PV after lyophilization and resuspension.

**Figure 5:**
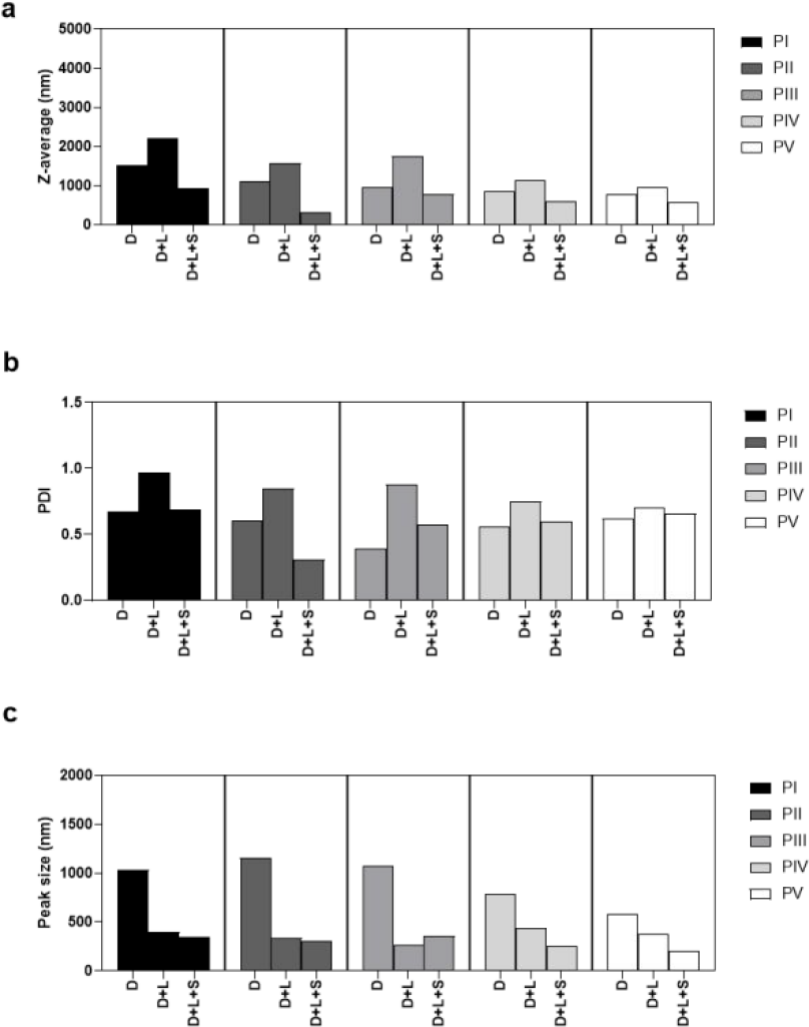
Particle sizes and size distributions of dialyzed samples. (a) *z*-Average particle size, (b) polydispersity index (PDI), and (c) main peak size of dialyzed (D), lyophilized and resuspended (D+L), or lyophilized, resuspended, and sonicated for 40 s (D+L+S) samples.

The *z*-average increased after lyophilization (P+L) and resuspension, for all samples (Figure 5). Especially, there was an increase in the PDI compared to the freshly precipitated (P) samples. This indicates that samples aggregated after lyophilization and resuspension. Tip sonication for 40 s was sufficient to break down these agglomerates, as shown by the *z*-average, PDI, and peak size curves leveling out (Figure 4). Comparison between the 40 s sonicated (P+L+S) samples and the freshly precipitated (P) samples show that the *z*-average and peak size values returned to approximately the same level as before lyophilization (Figure 3). This effect was most pronounced for the samples precipitated at the lowest concentrations, PIII–PV. For PI, the peak size prior to sonication was found to be inaccurately low due to severe sedimentation of the sample. As shown in Figure 4, the peak size of PI seemed not to change, although both the *z*-average and PDI decreased with the sonication time. This may indicate that the sample contained larger agglomerates that were not detected by the general-purpose method but that these were broken upon sonication. At the same time, the general-purpose model detected the smaller individual particles throughout the analysis, and these are represented by the peak size.

#### 3.2.3 Effect of Dialysis on the Particle Sizes and PDI

The particulate samples produced by precipitation contained 0.033–0.0033 M Na^+^ and Cl^–^ due to the reaction between equal mol diluted NaOH and HCl in the procedure, and the samples were therefore dialyzed to reduce osmolarity. Both the z-average and peak size of the samples (*D*) increased after the removal of ions by dialysis (Figure 5). This may be due to a combination of swelling and aggregation of the particles after several days suspended in water.

When the dialyzed samples were lyophilized and resuspended (D+L), both the *z*-average and the PDI were found to increase, as already observed for the ion-containing samples. This result may be another sign of swelling and aggregation during dialysis. Interestingly, the peak size of the dialyzed samples was found to decrease after lyophilization. However, another explanation is that the general-purpose model is poorly suited for these samples. Since this model works best for particle sizes below 1000 nm, larger agglomerates may not be detected. The *z*-average of ≥1000 nm as well as the high PDI values of the lyophilized samples were clear indications of severe aggregation and a poorly fitting model. After sonication, both the PDI and *z*-average decreased, while the peak sizes did not change much (D+L+S). This indicates breaking of agglomerates.

#### 3.2.4 Discussion of the Particle Characteristics

Visible inspection of the samples immediately after preparation showed that PI contained some large particles. These particles were sedimenting and therefore not detected by the DLS measurements. The peak sizes of PI were considerably larger than those of all other samples, which altogether indicated that the concentration used for PI yielded larger particles than the lower concentrations used. After lyophilization, PIII had a higher *z*-average than any other sample, although the peak size was within the range of the other samples. After sonication, the *z*-average was still high, around 1100 nm. This indicates that aggregation occurring during the lyophilization and subsequent resuspension was not broken up by sonication.

Otherwise, the samples did not seem to differ considerably in terms of particle sizes: all the samples PI–PV were within the 0.3–3 μm size range. The *z*-average was generally larger than the peak size, which might indicate the presence of particles too large for detection by the general-purpose distribution model. However, this may also be an allusion toward morphological heterogeneity within the samples. DLS measurements and calculations are based on spherical particles, and particles shaped differently may therefore be misinterpreted. The observed differences in z-average and peak size as well as the relatively high PDI values may indicate nonspherical particles. To gain insights into the morphology of the precipitated particles, SEM images were recorded of the dialyzed and lyophilized particles.

### 3.3 Particle Morphology

**Figure 6:**
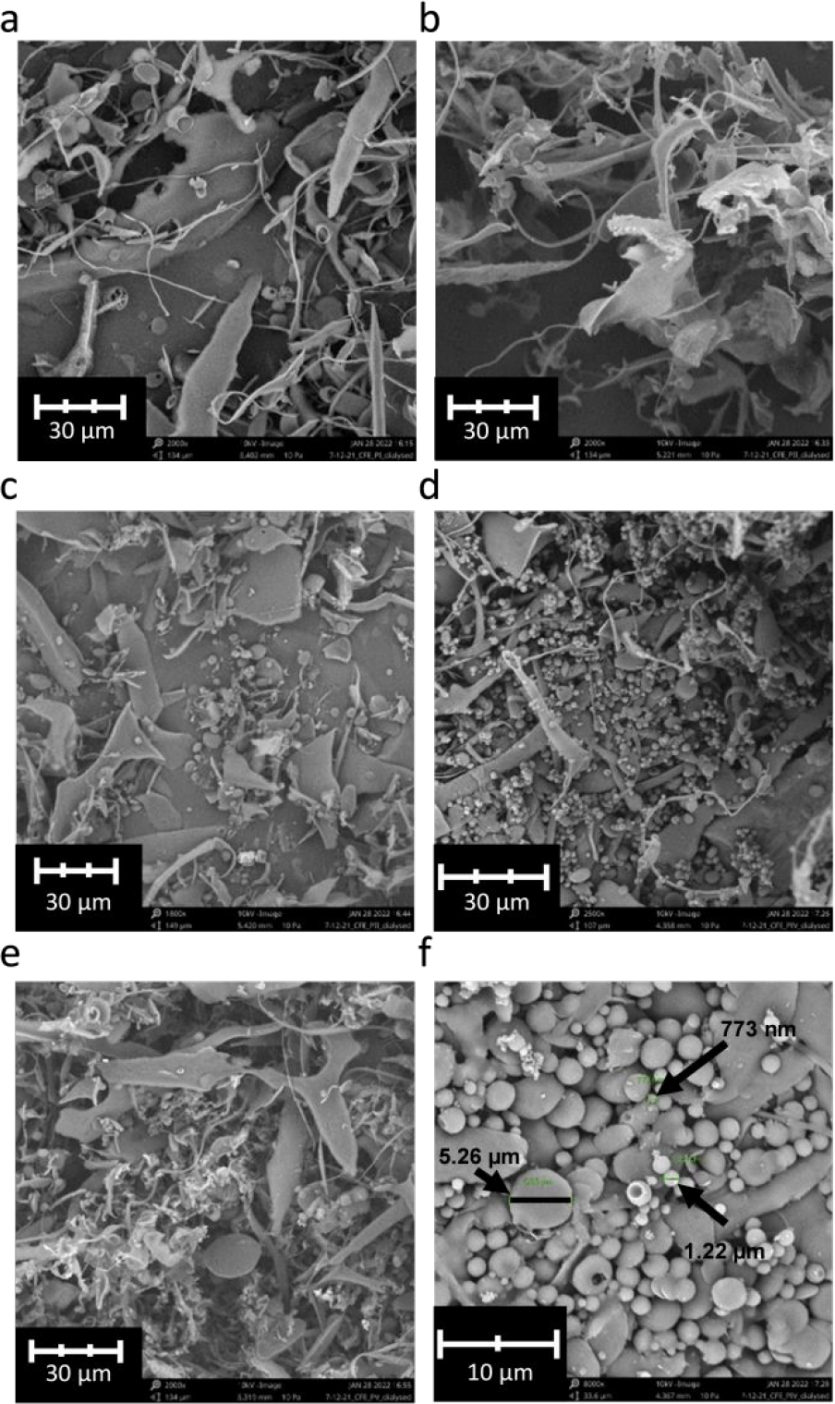
SEM images of dialyzed and lyophilized precipitation samples. (a) PI, (b) PII, (c) PIII, (d) PIV, (e) PV, and (f) close-up of PIV, showing size measurements of three particles.

All the dialyzed and lyophilized precipitated β-glucan samples, PI–PV, appeared morphologically heterogeneous, both between and within the samples, containing spherical particles as well as structures better described as strands and sheets (Figure 6a–e). PIV (Figure 6f) stands out as the sample with the highest amount of spherical particles. These particles appeared to vary in size, from <500 nm to >5 μm, whereas the majority of the spherical particles were around 1–2 μm in diameter. The larger particles appeared flattened compared to the smaller ones. This may be a result of the lyophilization process. It is currently not known why the precipitated β-glucans take up such different shapes. One possible explanation could be that the strands observed in these samples are derived from a longitudinal assembly of β-glucan triple helices. β-glucan-based particles prepared by precipitation have previously been described as an assembly of triple helices.^28^ Triple helix formation occurs mainly in aquatic solutions, while solvents such as DMSO and NaOH disrupt hydrogen bonds, and the polymers appear as random coils.^5^ Thus, DMSO or NaOH denatures the triple helices, while changing the solvent system by decreasing the NaOH concentration leads to renaturation.^36^ The ability to form triple helices depends upon the backbone structure and the branching degree. Both linear (1 → 3)-β-d-glucans and branched (1 → 3),(1 → 6)-β-d-glucans are known to form triple helices, but long branches are generally viewed as unfavorable for triple helix formation. Lentinan, schizophyllan, and scleroglucan are fungal β-glucans with a (1 → 3)-linked backbone and single glucose units attached at C-6 of approximately every third glucose residue of the backbone, which form triple helices in solution.^5^ Soluble lentinan has been shown to transition between triple helix and random coil conformation at a NaOH concentration between 0.05–0.08 M.^37^ Curdlan, a gel-forming linear (1 → 3)-β-d-glucan isolated from *Alcaligenes faecalis* var. *myxogenes*, has been shown to transition between the triple helix and random coils at NaOH concentrations between 0.1–0.3 M.^34^ AAoIw consists primarily of a (1 → 3)-β-d-glucan with occasional branching at *C*-6.^31^ Because the *A*_max_ of Congo Red solution changed slightly with the NaOH concentration, a series of blank solutions were prepared for comparison to the respective glucan solutions (Table 3). The results show that the *A*_max_ was shifted in the presence of AAoIw, which confirms triple helix conformation. The shift occurred at NaOH concentrations <0.3 M. In the presence of laminarin and BG-S, the *A*_max_ of the Congo Red was increased by 5 and 15 nm, respectively. High glucan concentrations had a slightly bigger shift in *A*_max_ compared to low glucan concentrations, which may indicate a certain inaccuracy as the glucan becomes very diluted. The results indicate that the triple helix conformation was formed at all the precipitation conditions used for preparing PI–PV (0.1–0.01 M NaOH). However, this also indicates that the morphological differences of PI–PV cannot be explained by differences in triple helix formation alone.

**Table 3:** Shift in Absorbance Maximum (Δ*A*_max_) of Alkaline Congo Red Solution in the Presence of *Albatrellus ovinus* β-Glucan

	Blank	AAoIW low		AAoIW high	
NaOH concentration (M)	*A*_max_ (nm)	Glucan concentration (mg/mL)	Δ*A*_max_ (nm)	Glucan concentration (mg/mL)	Δ*A*_max_ (nm)
0.30	485	1.5	0	3.0	+15
0.10	485	0.50	+10	1.0	+15
0.075	485	0.38	+10	0.75	+15
0.05	485	0.25	+10	0.50	+15
0.025	490	0.13	+5	0.25	+10
0.01	490	0.05	+5	0.10	+5

Another aspect related to the β-glucan concentration is the overlap concentration or critical concentration, which is when individual polymer chains are in contact and may interact with one another. For polysaccharides in solution, this typically affects their viscosity, as the viscosity will increase linearly with the concentration below the overlap concentration but exponentially above the overlap concentration.^38^ This is a result of a vastly increased possibility of intermolecular hydrogen bond formation. Similarly, for the particle formation during precipitation, it is possible that in the samples precipitated at the highest concentrations, there was extensive intermolecular hydrogen bonding, whereas intramolecular bonding was promoted as the concentration was decreased and the solution went from a semidilute to a dilute solution. This may in turn have promoted formation of spherical particles.

### 3.4 Binding to the Human Dectin-1a Receptor

**Figure 7:**
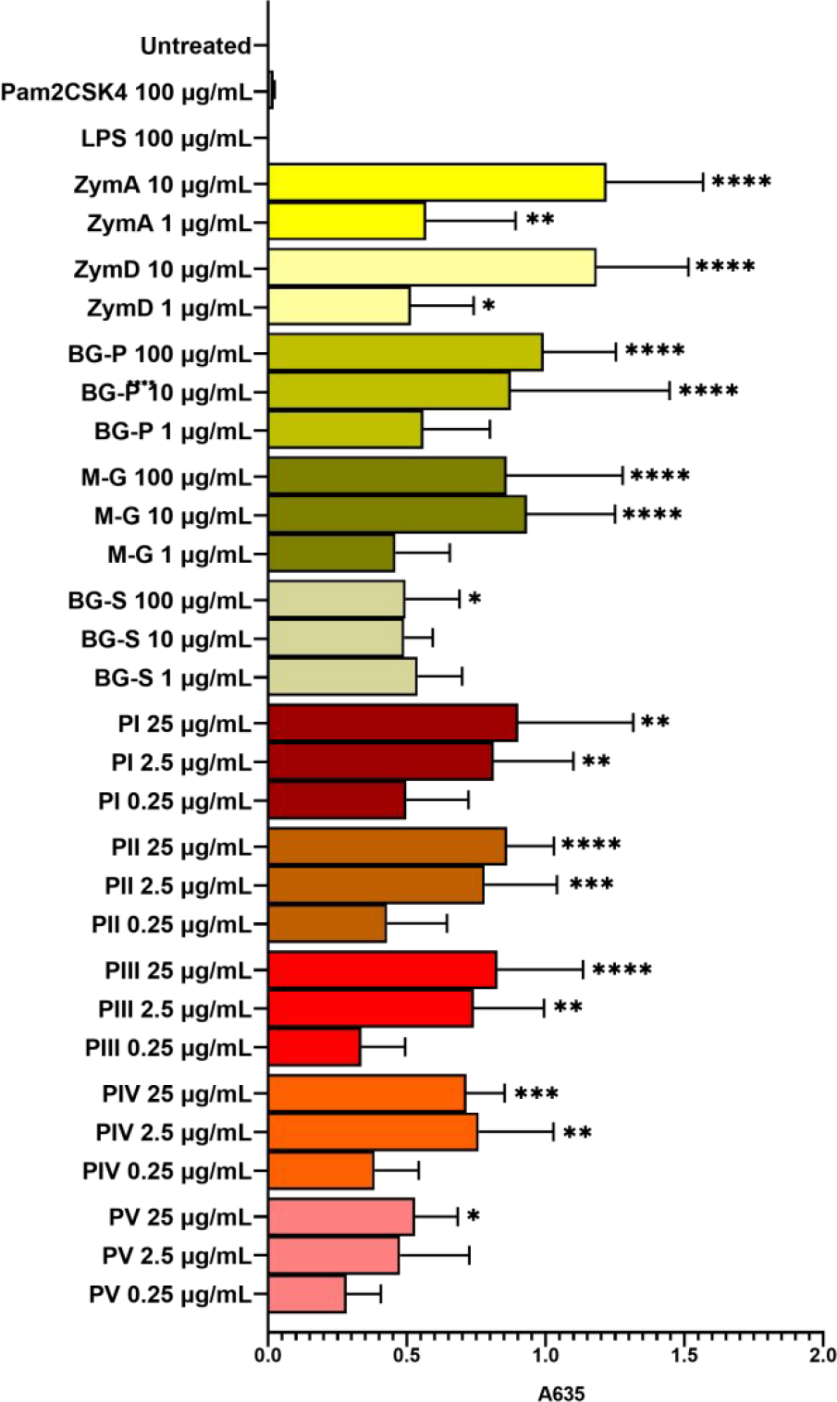
Binding to the human dectin-1a receptor on HEK-Blue hDectin-1a cells, as measured by cell supernatant absorbance reading at 635 nm. Error bars represent the SD of the three series of the experiment. LPS: lipopolysaccharide; ZymA: zymosan A; ZymD: zymosan depleted; BG-P: particulate β-glucan; BG-S: water-soluble β-glucan; M-G: M-Gard β-glucan; *P* = *A. ovinus* precipitated particles. * *p* < 0.0332, ** *p* < 0.0021, *** *p* < 0.0002, **** *p* < 0.0001, as compared to the untreated control.

The immunomodulatory activity of fungal β-glucans is achieved by activation of several different PRRs as mentioned above. Since dectin-1 is well-known to be involved in the immunomodulatory activity of particulate β-glucans, binding to this receptor may be indicative of such activity. Therefore, as an initial assessment of the samples’ interactions with the immune system, the prepared β-glucan particulate samples PI–PV were tested for binding to the human dectin-1a receptor using a reporter cell line model. The HEK-Blue hDectin-1a (www.invivogen.com) reporter cell line was used for this purpose. As these cells are not immune cells, they do not naturally express the dectin-1 receptor. Instead, these cells have been transfected with genes involved in the human dectin-1a/NF-κB pathway, thereby expressing dectin-1a. If stimulated with dectin-1a ligands, the cells produce a secreted embryonic alkaline phosphatase (SEAP). The detection medium contains a substrate for SEAP. When dectin-1a ligands are present, the cells secrete SEAP, and the substrate is converted into a product that can be measured by colorimetry. The cells did not respond to LPS but were found to respond to all the samples, PI–PV, as well as to the particulate β-glucan controls (Figure 7), in a concentration dependent manner. The activity of the samples was found to be comparable to that of the particulate yeast-derived β-glucans BG-P and M-G, and only slightly below the activities of ZymA and ZymD. The soluble BG-S was found to be less active toward the receptor, independent of the concentration range included in the assay. This is in accordance with previous results, where the soluble β-glucan laminarin, derived from the algae *Laminaria digitata*, proved to bind the human dectin-1a receptor but apparently to a smaller extent than the particulate yeast-derived β-glucans ZymA and ZymD.^39^ Although both the soluble and the particulate β-glucans bind the receptor, the response to the particulate samples was more intense than to the soluble ones. The dectin-1a receptor does not seem to differentiate significantly between the yeast and mushroom particles. BG-P had a mean particle size of 40 μm, with 80% of the particle population within the size range of 20–90 μm, while M-G had a mean particle size of 22 μm and a size range of 10–44 μm. Accordingly, both BG-P and M-G contained larger particles than PI–PV. ZymA has an average particle size of 3 μm.^40^ Both types of zymosan seemed to bind dectin-1a more than BG-P, M-G, and PI–PV. Samples PI–PV were closest in size to zymosan. Thus, the particle size does not seem to have a crucial effect on the receptor activity within the sizes included herein nor does the origin of the β-glucan. Whether the presence of various morphological structures affected the activation cannot be determined at this point due to the lack of knowledge on how the β-glucan-based particles behave in the medium. Simulation studies have shown that the presence of a triple helix conformation is favorable for a stable binding to the dectin-1 receptor.^41^ Presently, *A. ovinus* β-glucans were shown to form triple helixes in solution and bind to dectin-1 when in a particulate state. Based on the results, it seems likely that mushroom β-glucan-based particles have a potential as immune activators by binding to the human dectin-1a receptor, at similar levels as yeast-derived β-glucans. Further studies will evaluate whether the precipitation method can be utilized to prepare samples from other mushroom β-glucans and whether the dectin-1a binding properties translate to proinflammatory activation of immune cells.

## 4 Conclusion

The precipitation method described herein is a convenient method for the preparation of β-glucan particles, since it is rapid and does not require any special equipment, yielding particles around 0.3–3 μm in size, which is within the desired size range for macrophage activation and phagocytosis. The resulting particles were found to be heterogeneous in terms of size and morphology. Still, the precipitated samples PI–PV bound to the human dectin-1a receptor to a similar extent as commercially available particulate β-glucan preparations, which indicate a potential for use as immunomodulating substances. The precipitation method can thus be used as a rapid method to prepare dispersions from poorly water-soluble β-glucans, which can be used directly for *in vitro* testing of immunoactivity.
